# Using Schema Modes for Case Conceptualization in Schema Therapy: An Applied Clinical Approach

**DOI:** 10.3389/fpsyg.2021.763670

**Published:** 2022-01-20

**Authors:** David John Arthur Edwards

**Affiliations:** ^1^Department of Psychology, Rhodes University, Makhanda, South Africa; ^2^Schema Therapy Institute, Cape Town, South Africa

**Keywords:** schema therapy, schema mode, case conceptualization, schema perpetuation, mode sequences, blended modes, default modes

## Abstract

This article is situated within the framework of schema therapy and offers a comprehensive and clinically useful list of schema modes that have been identified as being relevant to conceptualizing complex psychological problems, such as those posed by personality disorders, and, in particular, the way that those problems are perpetuated. Drawing on the schema therapy literature, as well as other literature including that of cognitive behavior therapy and metacognitive therapy, over eighty modes are identified altogether, categorized under the widely accepted broad headings of Healthy Adult, Child modes, Parent modes and coping modes which are, in turn, divided into Surrender, Detached/Avoidant, and Overcompensator. An additional category is included: Repetitive Unproductive Thinking. This draws attention to the recognition by metacognitive therapists that such covert behaviors play a significant role in amplifying distress and perpetuating a range of psychological problems and symptoms. In addition to the modes themselves, several concepts are defined that are directly relevant to working with modes in practice. These include: default modes, blended modes, mode suites and mode sequences. Attention is also drawn to the way in which Child modes may be hidden “backstage” behind coping modes, and to the dyadic relationship between Child modes and Parent modes. Also relevant to practice are: (1) the recognition that Critic voices may have different sources and this has implications for treatment, (2) the concept of complex modes in which several submodes work together, and (3) the fact that in imagery work and image of a child may not represent a Vulnerable Child, but a Coping Child. The modes and mode processes described are directly relevant to clinical practice and, in addition to being grounded in the literature, have grown out of and proved to be of practical use in conceptualizing my own cases, and in supervising the cases of other clinicians working within the schema therapy framework.

## Introduction

Schema therapy is an integrative therapy that is increasingly being shown to be of value with clinically challenging presentations including moderate to severe presentations of personality disorders and complex trauma (Giesen-Bloo et al., 2006; Jacob and Arntz, 2013; Bamelis et al., 2014; Talbot et al., 2015; Keulen-de Vos et al., 2017; Peled et al., 2017; Huntjens et al., 2019; Simpson and Smith, 2020; Yakın et al., 2020; Bernstein et al., 2021). The identification and analysis of schema modes is central to schema therapy as a basis for case conceptualization and as a guide to practice. Schema modes are recognizable experiential states that express an underlying schema structure. The aim of this article is to present a comprehensive and clinically useful list of schema modes that have been identified in the clinical literature as relevant to conceptualizing psychological problems, and, in particular, to the way those problems are perpetuated, and that have proved to be of practical use in conceptualizing my own cases, and in supervising the cases of others.

## Schema Modes as Structural Units

The idea that there are parts of the self was already widespread in Europe in the second half of the 19th century. In 1868, Durand, whom Pierre Janet regarded as one of the founders of hypnotherapy, described how beneath the “ego-in-chief” was a multitude of “subegos” which were out of awareness and “constituted our unconscious life” (Ellenberger, 1970, p. 168). Berne (1961, p. 4), drawing on a concept developed by his mentor, Federn, used the term “ego-states” in his system of Transactional Analysis. He described three broad ego states, Parent, Adult and Child, which, as he pointed out, “are not just concepts but phenomenological realities,” that reflect real differences in the experience and behavior of the individual. This term is also the foundation of John and Helen Watkins’ ego-state therapy (Watkins, 1993; Watkins and Watkins, 1997).

There is increasing recognition that a range of distinct modes underpin everyday experience and behavior (Lazarus and Rafaeli, in press), and a growing number of current therapy approaches examine how to identify and work with them (Schwartz, 1997; Teasdale, 1999; Stiles, 2011). Referred to as schema modes within the schema therapy system, these “moment-to-moment emotional states that temporarily dominate a person’s thinking, feeling, and behavior” (Keulen-de Vos et al., 2014) are generated by the system of schemas that is the source of the “understandable patterning of cognition and affect that has developed from the past and structures current experience” (Stein and Young, 1997, p. 162). Each mode is based on an underlying “organizational unit” so that an individual can repeatedly exhibit the same cognitive, emotional and behavioral pattern (Lazarus et al., 2020). They are phenomenologically distinct: “each mode ‘feels different’,” observe Lazarus and Rafaeli (in press), with “distinct subjectively experienced qualities.” Dysfunction occurs when a mode, as “a part of the self” is “cut off to some degree from other parts of the self” (Young et al., 2003, p. 40).

## Differentiating Modes

When the concept of schema modes was introduced (McGinn and Young, 1996; Young et al., 2003) a relatively small number were identified and described. Since then, however, researchers and clinicians have been describing and naming an increasing number. Concern has been expressed at the apparent proliferation of modes. As Roediger et al. (2018, p. 42) point out, “if we use a close-up lens, we can distinguish an almost endless number of modes,” and this can be confusing for clinicians and clients alike. Lazarus and Rafaeli (in press), however, point out the need to find “the balance between optimal distinctiveness and parsimony.” As they observe, this depends on the context, and “we don’t know yet” just what that balance is for therapists engaging with complex cases.

Focusing on a limited number of modes can simplify case conceptualization and communication with the client. However, there are also disadvantages. Modes described in broad terms, may appear quite differently from one individual to another, or within the same individual at different times, or may include what are actually sequences of distinct experiential states. Consider Simpson’s (2020, p. 47) account of the ***Helpless Surrenderer*** mode, the heart of which is a feeling of being dependent, helpless, and needing to be rescued. The mode also includes, “a mixture of surrender and over-compensatory behaviors,” Simpson suggests, and “can have a resigned or victim tone… [and] can manifest with different ‘flavors,’ e.g., aggrieved, passive-aggressive, histrionic (theatrical, ‘flouncy’), sullen (‘teenager’), entitled, hopeless, negative, or complaining, under a façade of deference or submissiveness.” Furthermore, an item from the scale for measuring this mode (Simpson et al., 2018) includes a further feature, the hypervigilant attachment-seeking that may be a way of coping with abandonment: “I am very possessive and hold on to the people that are important to me.” Simpson is not describing a single, phenomenologically distinct experience, but a collection of modes that might occur together or in sequence. Such sweeping summaries under the heading of a single mode are not uncommon in the schema therapy literature.

## Modes and Their Relationships

There is consensus about the broad classification of modes under the headings of Healthy Adult, Child modes, Parent modes and Coping modes. Coping modes are divided into three subcategories: Avoidant/Detached, Overcompensator, and Surrender. There is some similarity between these categories and the coping styles identified by Horney (1945): moving away from, moving against, and moving toward others. However, there is not a perfect fit, and, as will be seen, there are some coping modes that do not fit clearly within any category system. Arntz et al. (2021) have recently suggested replacing the term Surrender with Resignation and Overcompensator with Inversion, however, the traditional terms will be used here.

First, a few other important concepts will be defined. The several modes or mode categories referred to will all be defined later. Emotion theory (Greenberg and Pascual-Leone, 2006; Elliott and Greenberg, 2007) makes a distinction between primary and secondary emotion. A Child mode has, at its center, a primary emotion, such as anger at unfair treatment, sadness at loss or disappointment, or fear in the face of threat. Primary emotions are adaptive when based on accurate appraisal of a current situation, as they provide guides to action. However, when a current situation triggers a schema based on past memories, primary Child emotions are evoked out of proportion to the present reality. These are, in effect, flashbacks and are maladaptive. Secondary emotions, by contrast, result from coping with (and blocking) a distressing primary emotion and are usually maladaptive. Within the schema therapy framework this means they are part of a coping mode and are not associated with vulnerability.

Modes often follow each other in recognizable patterns or **mode sequences** (Edwards, 2020). For example, a relationship conflict might activate a ***Vulnerable Child*** and an associated ***Parent mode***. This might be followed by a switch to a coping mode such as ***Compliant Surrender*** or ***Avoidant Protector*** or ***Scolding Overcontroller***, and this in turn might lead to a further mode. A client with bulimia nervosa who has been restricting her food intake (***Eating Disordered Overcontroller***) might experience intense craving and purchase a lot of food and eat it (***Detached Self-Soother***). After consuming it, she feels remorseful (***Shamed Child*** and ***Punitive Parent***) and determines to further restrict her food intake the next day (***Eating Disordered Overcontroller***). For a fuller example, see Edwards (2020).

Modes that are relatively stable over time are referred to as **default modes** (e.g., Lobbestael, 2008). Individuals who spend a lot of time in **default modes** appear to have a relatively stable personality, but this is often an ***Overcompensator*** and not the ***Healthy Adult***. Therapists can easily mistake a **default mode** for the ***Healthy Adult***, especially when it is, for example, a ***Social Overcompensator*** or a ***Pollyanna Overcompensator***. Therapists often need to help clients recognize that their default mode is a coping mode and not a fully adaptive way of being. By contrast, some dysregulated individuals display sudden shifts from one mode to another, referred to as **mode flipping** (Kellogg and Young, 2006). This is often confusing and disconcerting for those with whom the individual is interacting.

The term **Blended modes** refers to the way that features of more than one mode may appear together at the same time (Young et al., 2003). Blending is quite common and accounts for why, when we ask, “Which mode is the client in now?” the answer is not always straightforward. In the clinical literature authors often refer to child modes that blend separate distinctive experiences, for example, a ***Lonely Scared Child*** or an ***Abandoned-Abused Child***. Blending is also common in coping modes: a ***Self-Aggrandizer*** often blends with a ***Paranoid Overcontoller***, where self-righteousness and disdain are accompanied by a simultaneous wariness and suspiciousness. From this perspective, Simpson’s reference to “flavors” in the ***Helpless Surrenderer*** referred to above suggests how the mode can be blended with, for example, a ***Self-Pity Victim*** mode, or with a ***Complaining Protector*** or with a ***Defiant Child***. Another common blended mode is found in those who seek to ward off abandonment by pleasing the other person (***Compliant Surrender***) and making things perfect for them (***Perfectionist Overcontroller***).

I introduce the term **mode suites** as an extension of the concept of **blended modes,** to refer to a set of modes with similar or overlapping features which may blend and/or sequence quite smoothly. As I suggest below, the ***Healthy Adult*** is not a single mode but a suite of modes in which individuals act maturely. However, there is also a suite of modes associated with narcissism, where the individual seamlessly sequences between and/or blends, for example, ***Self-Aggrandizer***, ***Scolding Overcontroller***, ***Bully and attack***, ***Paranoid Overcontroller***, and ***Complaining Protector*** in a single stream of interaction. The range of modes and characteristics that may sequence or blend in this way is illustrated by Day et al.’s (2020) qualitative study of the experiences of individuals with a narcissistic relative.

**Backstage and frontstage:** Roediger et al. (2018) and Roediger and Archonti (2020) use the metaphor of a theater in which some modes are “frontstage,” clear and visible, while others are less easy to see and can be thought of “backstage” or even “offstage.” A coping mode (frontstage), for example, blocks out a Child mode that is being coped with (backstage or offstage). Nevertheless, as Behary and Conway (2021) observe, clinicians may be able to detect the message from the backstage Child mode that is “hidden behind a wall of survival” and “got lost in delivery.”

Thus, the primary emotions of a Child mode can be channeled through a coping mode. This can happen through **externalizing**, or **internalizing**. In externalizing, for example, anger, with its source in a Child mode (***Angry Child, Defiant Child***, or ***Enraged Child***), is channeled instrumentally toward others in a blaming and/or coercive manner through modes in which the aggression is a secondary emotion and is maladaptive (Edwards, 2021). These ***Externalizing Overcompensators*** will be described later. The primary anger can also take an internalizing, route. It may be directed into self-attacking (see the ***Flagellating Overcontroller***). However, when combined with helplessness it becomes a muted embitterment that can be redirected toward the self, providing justification for the self-pity of the ***Self-Pity Victim*** mode, from where it may be further externalized in ***Complaining Protector***. Or it may be redirected to keeping others out and resisting coercion, as in the ***Passive Resistor***, or (with further externalizing added) in the ***Angry Protector***.

## A Comprehensive List of Schema Modes

The recognition that modes may blend or sequence, points to the importance of recognizing a range of experiential states. Identifying and naming modes alerts clinicians to schema perpetuation processes, and failure to recognize them weakens the power of the case conceptualization. It is pragmatic to focus on a small number of prominent modes for therapists early in training, when therapists share the case conceptualization with clients, or for psychoeducational and experiential work with groups. However, with greater experience, particularly when working with long term clients, therapists’ development of a more differentiated capacity for recognizing modes sensitizes them to the range and complexity of modes and will enhance their ability to identify obstacles to progress and, as Lazarus et al. (2020) put it, “arrive at fruitful tailored formulations of their clients’ experiences.” The differentiation of modes is, as Lazarus and Rafaeli (in press) observe, at once “pragmatic” and “clinical” since it allows therapist and client to identify and communicate about a range of “experiential or agentic states.”

This is the rationale for the present categorization which includes over eighty different modes. It draws on several sources within the schema therapy literature (e.g., Young et al., 2003; Lobbestael et al., 2007; Bernstein and van den Broek, 2009; Flanagan, 2014; Dadomo et al., 2016) but identifies others that are described outside it. They are presented here under the familiar headings. After some reflection, I listed separately an important set of coping modes in the category ***Repetitive Unproductive Thinking*** **(RUT)**. Many of these are hard to classify under the headings of Detached/Avoidant, Surrender and Overcompensator for reasons that will be elaborated below.

I have largely used names already in widespread use. When working with clients, of course, therapists should use names that are meaningful to the client, rather than the formal names used here.

## Healthy Adult

The ***Healthy Adult*** is not a single mode but a suite of modes that can be identified in answer to the question, “How would a mature, compassionate and psychologically minded person think, feel and act in this situation?” Historically, we find conceptualization of mature functioning in Adler’s “community feeling” (Kałużna-Wielobób et al., 2020), Rogers’ (1961) “fully functioning person,” Maslow’s (1971) “self-actualization” and “self-transcendence,” and the traditional concept of “wisdom” (Baltes and Staudinger, 2000). Considerable attention has also been given to the dimensions of healthy functioning in the most recent editions of the DSM and ICD diagnostic systems and Bach and Bernstein (2019) have examined how these are consistent with, and help to elaborate, the concept of the Healthy Adult within schema therapy. Bernstein’s (2018) iModes cards draw on this vision of maturity. They pictorially represent 16 qualities of the ***Healthy Adult***, under four headings: self-directedness, self-regulation, connection and transcendence. Roediger et al. (2018) present a similarly expanded view. Against the background of this emerging consensus, these qualities are summarized here under seven headings which are intended to be practical with respect to assessing the degree of ***Healthy Adult*** qualities in a client as well as pointing to the kind of work needed to build or strengthen them^1^.

### Meta-Awareness: Capacity for Stepping Back and Reflection

Most therapy approaches recognize the importance of the capacity to step back and reflect on one’s own experience and that of others. Metacognitive capacity, referred to as “distancing” in Beck’s cognitive therapy, provides the basis for role-taking or perspective taking, an important capacity that emerges at the Piaget’s concrete operational stage as a result of what he called “decentring” (Beck et al., 1979). Deficits in the development of role-taking result in behavioral problems associated with impulsiveness and lack of reciprocity and training in role taking is included in many cognitive behavioral programs (e.g., Lochman et al., 2003). In psychodynamic approaches to problems arising from poor self-regulation, metacognitive capacity is called “mentalization” (Fonagy et al., 2004). In Acceptance and Commitment therapy, “defusion” (Roediger et al., 2018) literally means breaking the fusion between rational, reality-based cognition and irrational or distorted thinking patterns based in Child, Parent or Coping modes. In Transactional Analysis, Berne used the metaphor of “contamination” of the Adult, and emphasized the importance of undoing that contamination (Woollams et al., 1977).

The main focus in metacognitive therapy is on training clients to step back and note their own thoughts and reactions, to develop “detached mindfulness” (Wells, 2009). Training in mindfulness meditation also increases such metacognitive capacity. This promotes disidentification, the ability to step back from a part of the self and recognize it is “not me” or at least “not the whole of me.” This can lead to “deautomatization,” the gaining of self-control over previously automatic responses (Deikman, 1982). The term “disidentification” was also used in Psychosynthesis, a holistic psychotherapy developed by Assagioli who died in 1974. Teasdale (1999), a pioneer of mindfulness-based cognitive therapy, also refers to “decentering” and “disidentification.” van Vreeswijk et al. (2014, p.18) describe the integration of mindfulness with schema therapy, and point out its value in “turning off the ‘automatic pilot’,” and fostering “meta-awareness.” Metacognitive capacity provides the basis for clients to recognize and differentiate their schema modes, to view them self-compassionately and non-judgmentally, to reflect on their nature and function, and to deautomatize them. Without this, clients are “self-immersed” (Lazarus and Rafaeli, in press) and cannot usually experience any significant schema change.

### Reality Orientation

The Healthy Adult is oriented toward the realities of the world and has the capacity to make accurate, informed appraisals of everyday situations that are not distorted by simplistic thinking or jumping to conclusions that are not adequately based on information and evidence. This includes facing painful aspects of reality and not denying or distracting from them through coping modes. Associated with this is the ability to use information obtained to provide the basis for engaging in rational, and practical problem-orientated behavior, and responsibly performing the practical tasks that are part of effective living in all important life areas. This involves recognizing and identifying problems of all kinds, and taking steps so solve them in a mature way. This requires the skills of searching for and obtaining information relevant to understanding a problem, evaluating sources of information, evaluating options for action, and planning and evaluating the chosen actions (d’Zurilla and Nezu, 1999).

### Agency and Capacity for Taking Responsibility

***Healthy Adult*** maturity includes taking responsibility for one’s actions. This is associated with a sense of agency, feeling motivated and able to act in one’s own interests or according to one’s own values. This includes the capacity to make and keep commitments (Rogers, 1961). These are all aspects of integrity – being consistent and trustworthy, and acting with a clear moral compass that is grounded in an authentic sense of self. This is founded on metacognitive capacity because when individuals behave impulsively or automatically without reflection, they may have difficulty accepting responsibility for their actions. Flipping from one mode to another creates inconsistency. Individuals in a default coping mode such as an overcompensator are not fully in touch with their own needs and experience and the needs and experience of others; they may act consistently but usually in a manner that is inflexible and lacks integrity.

### Emotional Connection and Tolerance

The capacity to be humanly (emotionally) present and intelligently engaged with what has meaning and feels authentic is based on “openness to experience” (Rogers, 1961, p. 115). This means being in touch with one’s personal needs and emotional responses to situations and being able to tolerate the emotions experienced. Coping modes interfere with this, causing what Conway (2005, p. 606) calls “episodic inhibition,” that is inhibition of the part of autobiographical memory system that is connected to emotions and early schemas. The ***Healthy Adult*** has access to these emotions, but can exercise discrimination and self-control with respect to how needs, emotions and concerns are expressed. Openness to emotions also includes positive emotions, which get shut down by episodic inhibition, and allows in experiences such as gratitude and personal meaning.

### Reciprocity, Congruent Communication, and Democratic Values

The reciprocity principle is the basis for being able to engage in mature relationships in which there is mutual respect. This is at once a value and capacity. As a value, it means a commitment to having the same level of respect for the needs, perspectives, and experience of oneself and of others. As a capacity, it means being able act consistently on that basis. Combined with emotional connection and tolerance, this allows for congruent communication of experiences and needs, and mature reciprocity in interpersonal interactions and the ability to make respectful compromises in conflict situations. This is the foundation of assertiveness training, a well-established approach in CBT (e.g., Alberti and Emmons, 2008). The reciprocity principle is also the basis of democratic values toward the broader society in which one lives, with a capacity for acceptance and absence of prejudice toward others on the basis of ethnicity, culture, religious affiliation, political affiliation, sexual orientation etc.

### A Coherent Sense of Identity

An important Healthy Adult function is as an executive that integrates the functioning of the other modes (Young et al., 2003). This takes place as part of the normal, healthy development of executive functions. If that development is appropriately supported and not disrupted by unmet needs, trauma and neglect, individuals develop the capacity to sustain a coherent sense of who one is, with respect to personal beliefs, values, attitudes and motivations and feel a grounded sense of self that is consistent over time and through all significant life areas. This is accompanied by the capacity to accommodate a range of emotions and states, even when they are conflicting. Other people are experienced as separate and independent centers of their own experience. There is an absence of abrupt transitions between self-states or modes and an absence of dysfunctional modes in which the individual experiences extreme states, for example, of chaos, fragmentation or merger with another. Memory is largely accurate and consistent and not confabulated or combined with fantasy. The individual’s self-narrative (of who I am and what is important to me), is realistic and flexible and not marked by idealizations, oversimplifications, overcompensatory self-aggrandizement or self-identification as a victim (Horowitz, 2016). Impairment on this dimension links to Bach and First’s (2018) recognition of the importance of including an appraisal of severity in assessing personality disorder.

### Caring Beyond the Self: Compassion and Community Feeling

This refers to a sense of meaning and connection with life, a natural wisdom or spirituality that is not necessarily channeled through institutional religion, and which provides a sense of strength and direction in the face of loss and adversity. This engenders a sense of caring about and being motivated to strive for the common good, not just the good of oneself or one’s immediate family. Such individuals have self-worth based on what they have to offer, without needing to prove their worth and an attitude of kindness and compassion toward others, and spontaneous gratitude. They consider the effects of their actions not only from the immediate perspective, but also with respect to the effects for future generations. To this can be added the capacity for self-compassion (Neff, 2013).

## Dysfunctional Parent Modes

Dysfunctional parent modes are introjects, recordings, as it were, of the attitudes and behaviors of parents (or other authorities or caretakers) at times when they failed to meet the child’s core needs. Good parents who appropriately meet the needs of the growing child or adolescent are introjected as positive and functional, contributing to attachment security and adaptive adjustment. Dysfunctional introjects are reexperienced in the present as active voices (with for example punitive, shaming, demanding, guilt-inducing or anxiety-inducing messages), or as a felt sense of the parent figure and the implicit messages that s/he conveyed. Sometimes these introjects are projected on to the therapist, resulting in the client experiencing the therapist as likely to be, for example, unpredictable or punitive or making demands or disinterested.

The schema therapy literature mainly focuses on introjects that are experienced as explicit voices. These are now often called the Inner Critic modes. Roediger et al. (2018, p. 40) explicitly say that they were “formerly called parent modes.” While they are called dysfunctional parent modes by Farrell et al. (2014), Farrell and Shaw (2018) call them “dysfunctional critic modes” and point out that not calling them “parent” modes has the advantage that it does not trigger “defensiveness and family loyalty conflicts,” and does not complicate the inclusion of parents in the treatment of children and adolescents.

Here, they are still referred to as parent modes for several reasons:

(a) Introjection of the child’s experience of parents (or significant caretakers) is at the heart of these modes which correspond to what Bowlby called internal working models, which, as Roediger et al. (2018, p. 40) remind us, can go back to infancy.
(b) They are not just experienced as voices. With somatic focusing the experience of being shut out by a parent, or a parent being absent physically or emotionally (depressed parent, abandoning parent) can come into focus as a clear experience that is not like a voice.
(c) Being critical is only one aspect of failing to meet important childhood needs. Other behaviors such as neglect, overprotectiveness and permissiveness, contribute to the development of early maladaptive schemas (Peled, 2016).
(d) The term “parent” does not have to be used with the client. The term “critic” is appropriate where it captures the client’s experience of the mode. When, however, this is unmasked as originating from a particular parent, it may be useful to name it as a parent mode to bring the source of the introject into focus.
(e) Critical and demanding voices may not be introjects at all, as will be explained later.

Bowker (2021) recently listed 23 adjectives describing dysfunctional or toxic parents that can be introjected. In Table 1, 16 parent modes are differentiated and described under five broad categories. In practice, blended Parent Modes combine several of these within and across the categories.

**TABLE 1:** Dysfunctional parent modes.

**(1) Fails to give appropriate structure or guidance**	
*Neglectful*	Was not available enough, for example because the mother was overburdened with other children, depressed or busy with other activities. It is experienced not so much as the presence, but as an absence, as someone who may be physically present but out of reach when it comes to meeting important needs, and so implicitly conveying that “your needs are not important” (Peled, 2016).
*Indulgent*	Overly permissive, and indiscriminately permission-giving. Contributes to the development of a “spoiled” child who is entitled and lacking self-discipline (Peled, 2016). This may include one or more of: (a) *Overpermissive*: fails to set limits or to require self-control, (b) *Spoiling*: provides excessive access to material goods without expecting self-discipline or responsibility in return; (c) *Over-rewarding*: indiscriminately praises and expresses appreciation for the child’s behaviors regardless of whether they represent significant progress in developmental achievement.
*Naïve*	Immature, naïve, easily influenced by others, tends to accept people and circumstances as they are, fails to supply a sense of safety, teach right from wrong, or provide guidelines for anticipating consequences or handling everyday life situations (Peled, 2016). This might be a parent with a ***Pollyanna Overcompensator***.
**(2) Overprotects and interferes with the development of the child’s autonomy**	
*Overanxious*	Does not feel safe in the world and conveys that lack of safety to the child, implicitly and/or explicitly communicating that the world is not safe (Peled, 2016).
*Overprotective*	Encourages enmeshment and dependency, giving the message, “you can’t cope on your own,” “you can’t make your own decisions,” “You don’t know what you feel,” and “you depend on me and what I have to say in order to find out.” often blended with ***Overanxious Parent***.
*Victim* (Peled, 2016) or ***Guilt-inducing*** (Jacob et al., 2015).	Helpless, depressed, physically frail, and/or self-pitying. The implicit or explicit message to the child is that s/he is responsible for caring for the parent and for making him or her happy. In response, the child takes on adult responsibilities prematurely (a coping “parentified child”). May have been primarily in the internalizing ***Self-Pity/Victim*** mode or in the externalizing ***Complaining Protector*** mode, explicitly using his/her suffering to keep others emotionally tied to him/her (“emotional blackmail”).
**(3) Fails to attune to the child or breaks connection**	
*Invalidating*	Conveys to the child that what s/he is experiencing is not an accurate reflection of reality.
*Rejecting*	Rebuffs the child’s attachment-seeking behavior.
*Abandoning*	Suddenly unavailable, leaving the child alone. May be due to negligence or a family crisis such as parental illness including mental illness, e.g., depression (Cohn and Tronick, 1983), or from illness in the child resulting in hospitalization or some other form of separation.
**(4) Is critical and coercive**	
*Demanding*	Repeatedly pressures child to meet excessively high standards. Speaks with “should” and sets rigid rules and standards.
*Punitive*	Criticizes, threatens punishment and punishes in an unforgiving manner, explicitly telling the child that s/he is worthless and deserves punishment.
*Blaming*	Blames the child for specific behaviors or for being characterologically “bad.”
*Shaming*	Repeatedly shames and humiliates the child.
Coercive/controlling	Dictatorial, unilaterally issuing orders with the expectation that they will be followed and not challenged.
*Abusive*	Often in ***Bully and Attack*** mode, blending several of the modes listed above.
**(5) Is unpredictable**
*Chaotic/**Unpredictable***	Unstable, emotionally labile, unpredictable, disorganized, and often frantic. Is frightening and paralyzing for the child resulting in a state of constant hypervigilance. May be introjected as several split off parent modes that represent the different states the unpredictable parent displays (Peled, 2016, see also the drama triangle discussed below).

## Child Modes

Child modes are working models of the self, based on memories from childhood (including early childhood and infancy). These are encoded in the cognitive system called “implicational” by Teasdale (1993) and “episodic” by Conway (2005), which is directly connected to the brain’s emotional systems and is present at birth (and before). This is biologically separate from the language-based explicit system called “propositional” by Teasdale and “conceptual” by Conway.

### Vulnerable Child Modes

In ***Vulnerable Child*** modes, individuals are, in effect, re-experiencing states of vulnerability related to experiences of unmet needs in childhood. This broad category can be differentiated into experiences related to specific primary maladaptive schemas, referred to in the descriptions below. These are variously differentiated in the literature. For example, Young et al. (2003) refer to four: Abandoned, Abused, Deprived, and Rejected. Lobbestael et al. (2007) list three: Lonely, Abandoned/Abused, and Dependent, but add a Humiliated and Inferior Child as a subtype of Abandoned/Abused, a mode that is also included by Dadomo et al. (2016). Here eight distinct Vulnerable Child modes are identified, which can be combined into many blended Child modes.

***Lonely Child (Neglected Child):*** Feels alone with no one to turn to when faced with confusing or distressing experiences or situations. Parents have not been available to help the child with difficult emotions, so that the person feels empty, alone, socially unacceptable, undeserving of love, unloved and unlovable. ***Emotional deprivation, social isolation, defectiveness/shame schemas***.

***Abandoned Child:*** An intense experience resulting from sudden traumatic separation from the mother or primary caretaker. This can result in and engulfing experience of being all alone in an endless dark place when occurring in the first few months of life. ***Abandonment schema***.

***Rejected Child***: An experience of being emotionally cut off from a primary caretaker with an implicit or explicit message, “You are not wanted, I don’t care what you feel or what your experience is.” The child experiences invalidation of his/her needs and feelings. ***Defectiveness/Shame, Abandonment, Social isolation schemas**.*

***Terrified Child:*** An experience of intense terror in response to one or more traumatic events including exposure to or witnessing angry exchanges, threats or assaults from parents. ***Vulnerability to harm schema***.

***Abused Child***: Feels mistreated, abused, betrayed, and anticipates further abuse. There is usually a strong ***Punitive Parent*** voice. ***Mistrust/abuse schema***

***Humiliated/Shamed Child:*** Feels worthless and incapacitated by shame, anticipates further humiliation. ***Defectiveness/Shame schema**.*

***Dependent Child:*** Feels incapable of making own decisions, and believes that s/he needs a strong person at his/her side to guide him/her to make the right decisions. This is usually the result of overprotective parents who failed to encourage the development of autonomy and self-reliance. ***Dependence/incompetence schema**.*

***Desperate child:*** Feels desperate because of the intensity of the pain of basic needs not being met. May be related to any of the unmet needs so may blend several of the above. The pain is experienced as unbearable or close to unbearable and there is little or no expectation that those needs can be met or that the pain will end: often hidden behind a coping mode such as ***Helpless Surrenderer*** or an Overcompensator.

### Angry/Unsocialized Child Modes

These modes have their source in intense experiences of impulse or emotion from childhood that, if expressed freely, appear as immature and are often destructive to self and relationships.

***Angry Child:*** Experiences a deep sense of injustice, and feels anger about being unfairly treated, misunderstood, misrepresented, invalidated, dismissed or disregarded.

***Defiant Child:*** Angry response to having one’s autonomy curtailed and being told what to do, as might be expressed in the sentence, “I don’t want to, and you can’t make me!”

***Enraged Child:*** An experience of intense rage with an impulse to lash out and throw or smash objects in the environment or to retaliate against and hurt people, including physically assaulting and killing them (‘murderous rage’).

***Impulsive Child:*** Acts impulsively without the capacity for self-control or delay of gratification or regard for the possible consequences for self or others: “I want it and I want it now!” This mode is called the ***Impulsive/Undisciplined Child*** by Young et al. (2003). However, Lobbestael et al. (2007) and Roediger et al. (2018) separate out the ***Undisciplined Child*** which is a Coping child, as explained below.

***Spoiled/Entitled Child:*** Expects and feels entitled to have what s/he wants and not to have to exercise self-control or be concerned about the needs and reactions of others; often the consequence of a ***Permissive Parent***. ***Entitlement schema**.*

### Healthy Child Modes

These modes are healthy states which follow from the Child’s needs being met.

***Authentic Child:*** Experiences being in touch with and expressing one’s true self and a sense of authentic engagement with life. This is referred to as the True Self by Winnicott (1965/2016) who wrote, “Only the True Self can be creative and only the True Self can feel real.” An important goal of schema therapy is to restore and/or strengthen the individual’s access to this mode. Thus, Oldershaw et al. (2019) emphasize the importance of recovering a “lost emotional self,” without which an individual is like an orchestra without a conductor. Other writers use the metaphor of a compass (Rivera et al., 2019). This is the central Healthy Child mode: the terms below represent different elements within this broader experience.

***Contented Child:*** Experiences being at peace and satisfied because core emotional needs are currently met. Feels loved, contented, connected, fulfilled, valued, protected and safe. Although this is conflated with the ***Happy Child*** by Young et al. (2003) and Lobbestael et al. (2007), it seems appropriate to separate out this relatively passive contented state from more active energized ones.

***Happy Child:*** Experiences self-confidence, optimism, spontaneity and joy, a positive affectivity that is increasingly recognized as a feature of healthy well-being (Rivera et al., 2019; Harpøth et al., 2020).

***Playful Child:*** Experiences spontaneous playfulness and fun.

***Creative Child:*** Experiences a natural curiosity and creativity.

## Maladaptive Coping Modes

These modes are ways of coping with the emotional distress in the Child resulting from unmet needs due to toxic or neglectful parents, other caretakers, siblings and peers, or other traumatic and emotionally overwhelming events. Coping modes attenuate that distress, channel it (as when the ***Angry Child*** is expressed through an Externalizing Overcompensator, or block it out altogether). They result from a decision about how to cope that is usually implicit and not consciously planned (Edwards, 2019). Coping modes usually begin in early childhood, though they generally become elaborated in later childhood and adolescence. While most coping modes fit comfortably within the surrender, detached/avoidant and overcompensatory categories, some do not and I have separated out a fourth class of coping modes under the heading Repetitive Unproductive Thinking.

### Surrender Modes

The term “surrender” has three related meanings. First, it can refer to the way an individual surrenders to the underlying maladaptive schema, accepting the truth of schema beliefs such as “I am defective and unlovable” or “I cannot cope on own,” or “I will be let down and betrayed.” Coping involves acting in ways that seek to maintain connection with others in spite of these. Second, surrender can refer to submitting to the dominance of another person. In Compliant Surrender mode, both of these two meanings apply. Third, Young originally used the term schema “surrender” for states in which individuals behave as if they are like the child, with the same beliefs, emotions and behaviors as when the childhood pattern was set up. However, this is better conceptualized as a ***Vulnerable Child*** or an ***Angry/Unsocialized Child***, or a blend of these with a coping mode.

***Compliant Surrender:*** Acts from underlying subjugation, focusing on meeting the needs of others without attention to one’s own needs. Is subservient, self-deprecating, submissive, placating, and pleasing, toward others out of fear of conflict or rejection. Is likely to feel exploited and build resentment which may or may not be expressed (Lobbestael et al., 2007).

***Helpless Surrenderer:*** Feels helpless, dependent on others for help and in need of rescue (Simpson et al., 2018; Simpson, 2020). Passive, not expressing needs and not present in a vulnerable way. This seems to be coping by freezing with its origin in childhood experiences of coping with being overwhelmed by fear or other overwhelming emotions. As discussed above, other features that Simpson (2020) includes in this mode are better seen as other modes that sequence or blend with this mode.

***Self-Sacrificer/Rescuer:*** Focuses excessively on meeting the needs of others, with little attention to own needs. May be directed toward individuals or groups to whom one feels loyalty (family, a social organization, those that seem to be weak or in trouble or victimized (which may include animals as well as humans). This mode is often more satisfying and rewarding, at least in the short term, than Compliant Surrender, especially when blended with a ***Self-Aggrandizer***, but in the long term it can lead to burnout, and chronic fatigue. Many authors do not differentiate this from ***Compliant Surrender*** but phenomenologically it has several distinctive features and is part of the Drama Triangle, discussed below.

***Rolling Stone (passive):*** There is a lack of agency or capacity for commitment. It is named from the adage, “A rolling stone gathers no moss” (not from the music group) and is tapped by items in the Young Compensation Inventory: “I have trouble limiting myself to one job or career; I like to keep my options open,” “I like to be a ‘free agent’, to have the freedom to do what I want,” “I have trouble committing to one person or settling down.” It is constructed round an implicit acknowledgment of “I don’t really know what I want” and an underlying helplessness about that. In an overcompensatory version (described later), the individual takes pride in the freedom this provides and constructs it into an identity which belies the underlying helplessness and confusion.

***Passive-resistor***: Uncooperative and disinclined to take steps to engage in activities that would appear to others to be adaptive. There is likely a backstage ***Defiant Child*** expressed passively as opposed to actively (as in the overcompensatory ***Rebel*** mode). It is referred to as Stubborn Child by Dadomo et al. (2016), but is a coping mode not a child mode.

***Reassurance seeker***: Though often grouped as part of ***Compliant Surrender*** mode, reassurance seeking is an important behavior, in its own right, long recognized as significant in maintaining psychological problems such as OCD (Salkovskis and Westbrook, 1989), Health Anxiety (Salkovskis and Warwick, 1986) and other anxiety disorders. A scale has been developed to measure it (Rector et al., 2011). Reassurance-seeking can be a form of surrender to an underlying ***Dependence-Incompetence*** schema, and can sequence with ***Obsessive–Compulsive Overcontoller*** behaviors and various forms of rumination [thus Flanagan (2014) includes it under a “Worrywart” mode].

***Self-pity/Victim:*** Experiences self as a victim, whether of mistreatment by others or of circumstances, and responds with self-pity, expressed as “poor me” (Wessler et al., 2001; Edwards, 2020). Feels, helpless and passively awaits rescue while rejecting suggestions or help. There is often embitterment and resignation, coping with a ***Desperate child*** backstage. The individual appears distressed and even childlike, but there is no vulnerability (Edwards, 2015). See also Flanagan’s (2014) ***Victim***. This mode is particularly intractable when blended with a ***Care-seeking Overcompensator*** (see below) to create a ***Covert Martyr*** with an investment in the ***Victim*** role as a means of seeking validation for their sense of injustice about the way they have been mistreated (usually be their parents).

### Detached/Avoidant Modes

In these modes, individuals disconnect from the emotional distress associated with schema activation by avoidance. This can involve: (1) Emotional avoidance: automatic disconnecting from emotions (for example by shutting down the attachment system), (2) Behavioral avoidance: avoiding places, people, activities or conversation topics that might be triggering, or (3) Cognitive Avoidance: engaging in distracting activities that prevent distressing thoughts and emotions from coming to awareness.

***Detached protector:*** Withdraws psychologically from the pain of the EMSs by shutting off emotions, disconnecting from others, and functioning robotically. May remain quite functional in many practical areas of life. Usually has its origin in an early avoidant attachment to the mother (Young et al., 2003).

***Spaced out Protector:*** Shuts off emotions by spacing out or feeling sleepy. This can give rise to experiences of fogginess or unreality, and dysfunctional states of cognitive slowing and depersonalization.

***Avoidant Protector:*** Avoids triggering by behavioral avoidance of situations or cues that may trigger distress (Lobbestael et al., 2007).

***Deceptive Protector:*** Avoids telling the truth or tells half-truths (“white lies”) to avoid attracting blame or shame (Edwards, 2017). This is different from ***Conning and manipulative*** where the deceit is deliberate and strategic.

***Detached Self-Soother:*** Engages in activities that soothe, stimulate or distract from emotional distress. There are several categories of these behaviors that are often addictive or compulsive (Young et al., 2003).

(a) Excessive focus on adaptive behaviors such as working (workaholism) – occupational or domestic.
(b) Excessive time spent on unproductive activities that shut out unpleasant feelings; e.g., computer gaming, watching television, fantasizing, overeating (comfort eating), watching pornography, masturbating, taking ‘recreational’ drugs (self-medicating).
(c) Neutralizing: The compulsive behaviors found in OCD which are designed to neutralize disturbing intrusive images, impulses or thoughts (Salkovskis and Westbrook, 1989).
(d) Wanting to die (coping with a ***Desperate Child***) either passively or actively planning suicide.

Two other modes that can also be conceptualized as self-soothing are listed in other categories below. The ***Overcompensatory daydreamer*** is categorized below under **Repetitive Unproductive Thinking**. The ***Detached Self-Stimulator***, with its focus on risky and exciting behaviors is classified with the Overcompensators.

### Overcompensation Modes

In these modes, individuals act in ways that are opposite to the underlying schema they are coping with. These modes are misleading because they convey messages such as, “I am happy,” “Everything is fine.” “I am a warm and balanced individual,” “I am an interesting valuable person,” “I am capable and self-sufficient,” etc. that belie the underlying Emotional Deprivation, Social Isolation, Defectiveness/Shame, Failure, Dependence/Incompetence, etc. (Young et al., 2003). These modes often have an accompanying explicit narrative that supports the overcompensatory messages together with extensive rationalizations as to why they are true. On closer inspection, these narratives are delusional. The 48 items of the Young Compensation Inventory (YCI: Young, 1999) described a range of overcompensatory behaviors, and since then many more have been identified. Several of these features may combine to form **blended overcompensator modes**.

***Strong and Independent Overcompensator***: Individuals present a front of being strong, independent and capable and not needing support or assistance in any task they are engaged with or any problem they encounter. YCI item 20 reads, “I don’t like being dependent on anyone.” This mode often develops in childhood, where the child has to develop a premature independence, overcompensating for ***Dependence/Incompetence*** and ***Defectiveness/Unlovability*** and ***Emotional Deprivation*** schemas (a “parentified child”).

***Social Overcompensator***: Individuals present themselves as friendly and even warm, cheerful and happy. YCI item 44 reads, “I believe it’s important to “put on a happy face” regardless of what I feel inside.” But this is “fake happy,” a social façade that hides genuine feeling and experience, denies or minimizes problems and does not allow authentic engagement.

***Pollyanna Overcompensator***: Identified in people with eating disorders (Simpson, 2020), but by no means confined to them, this mode is optimistic, idealizing of family members who are, objectively, neglectful or abusive, and dismissive of the distress occasioned by events and conflicts that most people find painful. YCI item 43 reads, “I try to be optimistic at all times; I don’t let myself focus on the negative.” This is often achieved by means of platitudes like, ‘Everything happens for a reason,’ or ‘It was meant to be.” In this mode, individuals unwittingly invalidate their own or others’ struggles and difficulties.

***Comic Protector***: Makes jokes, or smiles and laughs as a way of avoiding sensitive topics entirely, or to distract and steer away from the emotions they activate when they arise in conversation.

***Rolling Stone:*** In contrast to the Surrender version [***Rolling Stone (passive)***], the individual takes pride in the freedom provided by failure to commit and constructs it into a positive identity which belies the underlying helplessness and confusion. This is captured to some extent by the YCI item, “I like to be a ‘free agent,’ to have the freedom to do what I want,” which is likely accompanied by rationalizing self-justification about why this is a healthy choice. Can be blended with the ***Rebel*** mode (see below).

***Attachment-seeking Overcompensators:*** Modes through which individuals seek to gain or maintain connection with another in a manner that is coercive and sometimes entitled.

(a) ***Attention and Admiration Seeker:*** Engages in inappropriately extravagant, dramatic and exaggerated behavior (including sexually seductive behavior) to impress others and receive attention and admiration: called Attention and approval seeker by Lobbestael et al. (2007).
(b) ***Hypervigilant Clinger:*** Attempts to prolong contact, refuses to leave, clings, or even begs in response to impending separation (Behary, 2014). May blend with ***Scolding Overcontroller***.
(c) ***Care-seeking Overcompensator:*** Individuals act disarmingly, with appealing charm, helplessness or present as sick, in a manner that coerces others to provide care (Wilkinson, 2003). Despite the apparent helplessness, in the interests of secondary gain, they exert pressure on others who experience them as coercive and frustrating and the care may include being admitted to hospital or some other place where they will be attended to.

***Detached Self-Stimulator:*** Excessive engagement in activities that are risky and exciting such as gambling, dangerous sports, promiscuous sex. This thrill-seeking is classified as an overcompensator since the overt behavior belies the underlying emptiness.

***Overcontrollers:*** These protect from perceived or real threat by focusing attention on detail, and exercising extreme control (see Bamelis et al.’s 2014 ***Controller*** mode).

(a) ***Perfectionistic Overcontroller:*** focuses on getting things perfect to attain a sense of control and safety and ward off misfortune and criticism (Lobbestael et al., 2007). YCI item 8 reads, “I work hard to be among the best or most successful.” The significance of unhealthy or “clinical perfectionism” has long been recognized in CBT and there are several existing measures of it (Shafran et al., 2016).
(b) ***Eating Disordered Overcontroller, or Anorexic Overcontroller:*** an elaboration of the ***Perfectionistic Overcontroller*** with a focus on controlling body mass and becoming/remaining thin. Relentlessly applies perfectionistic and usually unrealistic about goal weight and foods that must be avoided (Edwards, 2017, 2020; Simpson, 2020).
(c) ***Invincible Overcontroller*** (Simpson, 2020): Individuals believe and act as if they are omnipotent and can achieve anything they want by sheer determination and force of will (American Psychological Association [APA], 2021; Kernberg, 1995). This can blend with a ***Self-aggrandizer***, but may, by itself, lack the competitiveness and disdain of that mode and rather blend with a ***Strong and Independent Overcompensator***.
(d) ***Obsessive–Compulsive Overcontroller:*** suppresses uncomfortable feelings by giving attention to detail or neutralizing them with repetitive ritualistic behaviors which may be overt (repetitive washing or checking or tidying and cleaning the house) (Bernstein and van den Broek, 2009). This is tapped by YCI item 8, “I put a lot of emphasis on having order in my life (e.g., organization, structure, planning, routine).” Covert neutralizing (such as repeating words or phrases) is classified below under Repetitive Unproductive Thinking.
(e) ***Suspicious Overcontroller*** (or ***Paranoid Overcontroller***): anticipates that others will be malevolent, betray, and want to harm him/her, and is overly sensitized to seeing evidence for these things and vigilantly scans for them (Lobbestael et al., 2007). This is typically a way of coping with an ***Abused Child*** with a ***Mistrust/abuse*** schema.

***Externalizing overcompensators:*** These modes overlap to some extent and the features may blend or sequence.

(a) ***Self-aggrandizer:*** Behaves in an entitled, competitive, grandiose, or status-seeking way (Young et al., 2003; Lobbestael et al., 2007; Behary, 2013). This is reflected in several YCI items including 17: “I like being in positions where I have control or authority over the people around me.” The individual is self-righteous, boastful, self-absorbed, lacks empathy, does not believe s/he should have to follow rules that apply to others and expects to be treated as special.
(b) ***Angry Protector:*** Uses a displays of anger to keep others at a distance and protect him/herself from others who are perceived as threatening (Lobbestael et al., 2007). The anger may be muted or partially suppressed, but the mode may sequence with other externalizing overcompensators such as ***Bully and Attack*** or ***Complaining Protector.***
(c) ***Scolding Overcontroller*** issues orders to others in a domineering way and makes belittling remarks as a way of controlling their behavior (Edwards, 2013, 2015); usually in a suite with a ***Self-Aggrandizer.***
(d) ***Bully and Attack:*** The individual actively attacks and seeks to hurt others, demeaning and humiliating them in a controlled, strategic and sadistic way. Called the ***Bully*** by Flanagan (2014), it can involve verbal and physical abuse or threats, sexual coercion and antisocial and even criminal acts (Lobbestael et al., 2007; Bernstein and van den Broek, 2009).
(e) ***Complaining Protector:*** Individuals feel victimized and embittered, and vent their anger in a constant stream of complaints, directed at other people or institutions or the world in general, or at the person they are talking to. This is an externalizing version of the ***Self-pity/Victim*** mode, sometimes called the ***Help-rejecting complainer*** (Edwards, 2015) because any attempt to offer advice or help is ignored or dismissed (Bernstein and van den Broek, 2009). When blended with a ***Self-aggrandizer*** individuals boast about the degree to which they have suffered and feel entitled to be listened to and sympathized with (Behary and Conway, 2021). When blended with a ***Self-sacrificer/Rescuer***, the individual induces guilt (“after all I have done for you”) to create a ***Martyr*** mode (Behary, 2021) which we might call an ***Overt Martyr*** to distinguish it from the ***Covert Martyr*** described above.
(f) ***Rebel:*** This is tapped by several YCI items such as “I dislike rules and can get satisfaction from breaking them,” “I think of myself as a rebel; I often go against the established authority,” and “I enjoy being unconventional, even if it’s unpopular or I don’t fit in.” This mode externalizes a backstage ***Defiant Child***, and incorporates a narrative that portrays it as a valuable identity. Expression may range from being more passive-aggressive to being actively, even militantly rebellious. Flanagan (2014) names a ***Rebel*** mode but does not fully differentiate it from the ***Defiant Child***.
(g) ***Conning and Manipulative:*** An extreme form of overcompensation that results in abusive and even criminal behavior in which the individual cons, lies, or manipulates in a manner designed to achieve a specific goal, which either involves victimizing others or escaping punishment (Lobbestael et al., 2007; Bernstein and van den Broek, 2009).
(h) ***Predator:*** Another extreme form of overcompensation that results in abusive and even criminal behavior in which individuals focus on eliminating a threat, rival, obstacle, or enemy in a cold, ruthless, and calculating manner (Lobbestael et al., 2007; Bernstein and van den Broek, 2009).

### Repetitive Unproductive Thinking Modes

In these modes, individuals become caught up in repetitive rehearsal of thoughts and/or images in a manner that does not result in productive planning or problem-solving, and that perpetuates or even exacerbates problems. These modes all involve covert or largely covert processes that have a lot in common among themselves and have been extensively documented in the cognitive-behavioral literature where they are often referred to as repetitive negative thinking (RNT: Ehring and Watkins, 2008). I call them “unproductive” rather than “negative” because some of them, like the ***Overcompensatory daydreamer***, are less obviously “negative” in the moment even though they contribute to perpetuating problems.

Like all coping modes, they shut out access to authentic feeling in the Child and the emotions associated with them are secondary, rather than primary (Elliott and Greenberg, 2007). Many of these modes do not fall neatly under the categories of Surrender, Avoidance and Overcompensation, because they involve sequences of submodes. For example, depressive rumination or worrying may start with triggering of a primary schema such as Defectiveness, Abandonment or Vulnerability to Harm. The theme of the schema is then projected into a present situation and elaborated with cognitive distortions which are repeatedly rehearsed. This rehearsal keeps activating the schema which maintains the brooding on painful scenarios and this may alternate with futile attempts at problem-solving. These latter activities block access to the ***Vulnerable Child***.

In the schema therapy literature, Edwards (2013) referred to a ***Worrying Overcontroller***, in a case study, adapting the insights of Borkovec et al. (1998) into the language of schema modes. Brockman and Stavropoulos (2020) and Stavropoulos et al. (2020) examine many of these modes and refer to them as ***Overanalyzers***. This term captures the phenomenology of only some of these modes and is included in the categorization below. Effective treatment needs to include a focus on developing meta-awareness (Wells, 2009; Montgomery-Graham, 2016; Watkins, 2016; Topper et al., 2017; Hvenegaard et al., 2020), and for the schema therapist, the challenge is to use meta-awareness to unmask and by-pass the RUT coping to connect to the primary vulnerability.

I categorize them under five broad categories that focus on, respectively, (1) self-doubt, (2) denial of reality, (3) anticipating that one will not have the resources to cope with threat, (4) brooding on defectiveness, abandonment and loss, and (5) anger. More specific forms of rumination that have been described in the literature can be classified within these broad categories. However, some may contribute to more than one. For example, some of these listed under ***Worrying Overcontroller*** can exacerbate depression too.

(1) ***Overanalyzer:*** This mode is characterized by rehearsal of thoughts that focus on self-doubt, self-questioning and questioning the motivation of others or broader “Why?” questions about life and its meaning. This is often referred to as “second-guessing.” There may also be repeated attempts to mentally reassure oneself and attain certainty in an uncertain world. This is done by repeatedly reviewing and checking some aspect of one’s behavior to see whether it meets a standard or whether it was well received by another person. We know that reassurance-seeking as an external behavior does not work: the person seeks reassurance about something from another person and when they receive the reassurance they feel better but only briefly. Soon the self-doubt and self-questioning return. The same applies for reassurance-seeking that is entirely covert. Even if the person gets to the point of feeling reassured, it does not last, and the rumination continues.
(2) ***Denial Ruminator:*** In contrast to most other forms of rumination, these forms are particularly effective in damping down or shutting out the painful emotion they are coping with.
  (a) ***Counterfactual Ruminator*** engages in repetitive regretful thinking about what would have happened “if only” one had acted differently, or some other condition had been different. It is counterfactual in that the individual is imagining what it would be like if the facts were different (Tanner et al., 2013; Mitchell et al., 2016). This “if only …” thinking, with a focus on how a traumatic event might have been prevented, is a significant factor in preventing emotional processing of a traumatic event, and in perpetuating PTSD (Clark and Ehlers, 2005; Michael et al., 2007; Turliuc et al., 2015). When the event is the death of a loved one it is often called ***Grief Rumination*** (Smith and Ehlers, 2020). Traditionally this is called “undoing,” because the individual is trying to undo a reality that cannot be undone.
  (b) ***Overcompensatory daydreamer:*** Spends time on overcompensatory daydreaming about, for example, finding the man/woman of one’s dreams, experiencing ecstatic sex or becoming successful, wealthy or famous, in the absence of any realistic plan likely to lead to achieving such goals. Although an overcompensation, individuals in this mode are passive and the mode can also be considered a form of ***Detached Self-Soother***.
(3) ***Worrying Overcontroller:*** In this mode, individuals ruminate on their inability to cope with a variety of situations and what is likely to go wrong as a result. Over 30 years ago, Borkovec recognized this as a form of coping that “functions as a type of cognitive avoidance, and inhibits emotional processing” (Borkovec et al., 1998, p. 561). Anxiety is a common response to facing a situation which one does not believe one has the resources to cope. Consequently, repetitive thinking, with the focus on not being able to cope, generates chronic anxiety and contributes to Generalized Anxiety Disorder. Some people with this mode believe that worrying helps them cope better and that they need to do it to protect themselves. Others, by contrast, are concerned about not being able to control the worrying and worry further about that.
  (c) ***Event Post-Mortem Ruminator***: Following a social interaction, individuals repeatedly review aspects of their own behavior focusing on evidence that they did not perform well, made a poor impression on others or were humiliated. This mode has been described and targeted in cognitive therapy for social anxiety (e.g., Clark and Wells, 1995; Hackmann, 1997).
  (d) ***Catastrophiser:*** Individuals ruminate on, and may actively visualize, the worst possible outcomes that might occur. This follows triggering of a ***Vulnerability to Harm*** schema and the worrying repeatedly reactivates it.
  (e) ***Covert Obsessive–Compulsive Overcontroller***: The individual repeatedly (and sometimes desperately) rehearses images or thoughts in an attempt to neutralize other distressing images or thoughts: for example, ritualistic counting or praying or “thinking a good thought to get rid of a bad thought.” Like overt behavioral compulsions, these activities are overcompensatory.
  (f) ***Body checker:*** A largely covert form of obsessive–compulsive behavior in which individuals spend many hours a day checking their appearance. A common means is a mirror or another reflective surface. Using a car mirror can result in being late for work or social events or cause traffic accidents. Those with Body Dysmorphic Disorder focus on body parts whose appearance they are concerned about – “hot spots,” a behavior that maintains preoccupation with and shame about one’s appearance (Veale and Riley, 2001). In Anorexia Nervosa and other eating disorders, body checking can involve pinching the skin at the abdomen to check for body fat, pressing the skin to feel one’s bones, mirror checking in which body parts are scrutinized to check if they are thin enough. This is often sequenced with a ***Flagellating Overcontroller*** (see below) self-talk – “look at those disgusting scars,” “my nose is just gross,” “look at those fat rolls,” that focus on perceived defects (Simpson, 2020).
  (g) ***Threat image ruminator:*** The individual is preoccupied with spontaneous threat-related images (visual, verbal, somatic) that are fear related and arise from the ***Terrified Child*** (or ***Terrified Adult*** if related to a more recent trauma). Such images are a feature of PTSD where, in addition to flashbacks to traumatic moments, images linked to cues at the time of a traumatic event can be taken as warning signals that something threatening is about to recur (Ehlers et al., 2002). Examples would be, for someone involved in a vehicle collision at night, seeing headlights of a car rapidly approaching, or, for an abuse or torture survivor, hearing footsteps that preceded the arrival of the abuser. Other images may be “linked to preoccupations with appraisals of the trauma and its sequelae, rather than representing trauma *memories*” (Ehlers et al., 2002, p. 1000). Such images are also common in eating disorders where the fear of becoming fat is portrayed in images of parts of the body covered in folds of fat. Simpson (2020) attributes these images to a ***Punitive Parent*** mode, but, whatever the source, they become a focus of preoccupation and rumination.
(4) ***Pessimistic*** or ***Depressive Ruminator:*** Focuses on repetitive thoughts related to defeat, despair and hopelessness about ever being able to address schema-based experiences such as a sense of unworthiness (***Defectiveness/Shame*** schema), rejection and abandonment (***Abandonment*** schema), social unacceptability (***Social isolation*** schema), failure (***Failure*** schema), or incompetence (***Incompetence/Dependence*** schema). Considerable attention has been given to this by Papageorgiou and Wells (2004) and Watkins (2016).
  (h) ***Social Comparison Ruminator*** engages in repetitive comparisons of oneself with others with respect to such aspects as appearance, body mass, practical, professional or social skills. This may be associated with a form of ***Counterfactual Rumination*** that focuses on, “If only I was thinner… had shinier hair … had these skills. had gone on a course about … etc.” This amplifies beliefs and feelings related to schemas such as defectiveness, failure and incompetence.
(5) ***Angry Ruminator***: In this mode individuals ruminate angrily with a focus on having been mistreated in various ways: let down, neglected, abandoned, scorned, betrayed (Karantzas, 2021). Rather than immediately channeling the anger through an ***Externalizing Overcompensator***, they repeatedly rehearse thoughts about the mistreatment, the unfairness of it, and the speculate on the motives of the person they perceive as having mistreated them (Sukhodolsky et al., 2001). Two specialized Angry Ruminators are:
  (i) ***Vengeful Ruminator***: Individuals rehearse vengeful thoughts and fantasies toward individuals they believe have harmed or traumatized them. This perpetuates many psychological problems including PTSD (Gäbler and Maercker, 2011; Rowe et al., 2018).
  (j) ***Flagellating Overcontroller:*** This is a voice that is self-attacking and shaming (Simpson, 2020, p. 51). It is also self-motivating, but in a harsh and cruel manner. It is a harmful and often intractable form of rumination. For a measure related to this, see Smart et al. (2016). As Simpson points out, it is not a ***Punitive Parent*** (not an introjected recording of a Parent), but is the individual’s own ***Angry Child*** – or ***Enraged Child*** - that is internalized, attacking the self instead of those who the child is really angry with (e.g., neglectful or abusive parents). As Simpson states, “The anger is re-directed inwardly to protect the stability of the wider family system.” For further discussion of the differentiation of this mode, see the section below on Sources of Critical and Demanding Modes.

## Mode Differentiation: Some Further Challenges

In conclusion, seven other concepts and processes will be examined that complicate the process of differentiating and working with modes.

(1) The Coping Child: Most coping modes have their origins in childhood, some in very early childhood or infancy. A Coping Child refers to a child who is in a coping mode. It is important to recognize this when working with imagery because, when the client has an image of a child, it may be a Coping child (Edwards, 2019) or a Protector Child (Edwards, 2020, p. 270), rather than a ***Vulnerable*** or ***Angry Child***. Since Child modes are the site of primary emotions while coping modes are the site of secondary emotions, a coping mode that presents as childlike is not, technically a ***Child*** mode, but a coping mode, developed in childhood to cope with intense experiences of unmet needs. In deep experiential work, we often find that current coping modes have been there since early childhood and incorporate quite simplistic and childlike beliefs and perspectives. When they appear in adults, it is easy to lose sight of their developmental origins, as these modes become elaborated with resources from later developmental periods. Most coping modes may have an underlying **Coping child** origin. Two examples are given here.
  • ***Parentified Child***: This is a child who, in response to a ***Guilt-inducing Parent*** or ***Victim Parent*** (see above) feels over-responsible for the well-being of the other, focuses on the needs of other, feels guilty if s/he fails to keep the other happy, and behaves in a prematurely independent way. This Coping Child is coping with the deprivation and guilt by subjugating his/her own needs (***Compliant Surrender***) and switching into a ***Self-Sacrificer/Rescuer*** mode, often blended with a ***Strong and Independent Overcompensator*** and a ***Perfectionist Overcontroller***. This mode, which may be evident in children (the context in which the term originated), can also be recognized when bridging back to childhood memories in someone with the coping modes just referred to.
  • ***Undisciplined Child***: Although sometimes conflated with the ***Impulsive Child*** (e.g., by Young et al., 2003), the ***Undisciplined Child*** and ***Impulsive Child*** are different. The ***Impulsive Child*** is a genuine child mode, while the ***Undisciplined Child*** is a coping mode or a sequence of coping modes. In this mode, the individual “cannot force him/herself to finish routine or boring tasks, gets quickly frustrated and soon gives up” (Lobbestael et al., 2007, p. 84; see also the ***Stubborn Child*** of Dadomo et al., 2016). This can contribute to procrastination, beginning, for example, with a primary experience of frustration or defiance, followed by an experience of ***Helpless Surrender*** and end with a coping mode such as ***Detached Protector***, ***Avoidant Protector***, ***Detached Self-soother***, ***Passive-Resistor*** or, ***Complaining Protector***.
(2) **Simple and composite modes:** We can distinguish between simple modes that have few distinctive elements and composite modes that have many. In ***Detached Protector***, for example, individuals are emotionally disconnected and robotic in a manner that is simple, consistent and recognizable. However, in some modes, several elements work together, some of which can themselves be identified as modes. These function as sub-modes within the larger system of a composite mode. For example, Edwards (2017) showed how an ***Eating Disordered Overcontroller*** included the following elements: (a) a decision to lose weight and get thin in order to feel in control and to become more attractive, popular and lovable; (b) a set of rules or standards to be followed with respect to diet and how parts of the body should appear, (c) a vigilant focusing of attention on situations where those rules or standards are to be applied, and behaving in a manner that follows from that; (d) motivational activity in the form of self-instructions and self-criticism that encourage the individual to maintain the standards, and punish the individual if the standards are not met (a ***Flagellating Overcontroller***); (e) a threat-warning mode that generates images of the body with large deposits of fat in various areas, to deter her from breaking the food rules; (f) a ***Rumination*** mode that is preoccupied with repeatedly checking her body and her diet with respect to whether they comply with the standards set. The way these various elements work together to implement the decision that created the mode in the first place show how a composite mode is more than just a habitual mode sequence.
(3) **Parent–Child mode dyads:** There is a dyadic relationship between a maladaptive ***Parent*** mode and a ***Vulnerable***, or ***Angry Child*** mode. When a trigger situation activates a ***Child*** mode, the ***Parent*** mode with which it is dyadically connected is activated too (Edwards, 2017, 2019). The working models of ***Child*** and ***Parent*** are not independent of each other and form a ***Parent–Child mode dyad***. This follows from Bowlby’s account of how attachment patterns involve working models of self and other in relationship. Four decades ago, Kernberg called these dyads “units” which are “constellations of affective memory” which combine an experience of self, and experience of other, and an affective charge (Christopher et al., 2001, p. 691; Clarkin et al., 2007). In schema therapy, this is recognized by Rafaeli et al. (2011, p. 64) and by Dadomo et al. (2016), who observe that, “the Punitive/Critical parent mode and the Vulnerable Child mode exist in a victim-abuser relationship to one another, with the critical, punitive voices triggering painful feelings of worthlessness and depression.” However, phenomenologically, it is difficult to ascertain a temporal sequence. If someone, hearing an angry remark, is triggered into a ***Defective Child*** mode, the introjected ***Blaming*** or ***Shaming Parent*** is activated simultaneously.
(4) **Mode cycles:** These are sequences that take place between two individuals where the behavior of the one triggers the other, who in turn behaves in a manner that triggers the first and this leads to a self-perpetuating cycle that creates chronic disconnection. This concept is central to emotionally focused therapy for couples (Johnson, 2004), where the members of the couple are invited to step back and see the cycle and recognize that the enemy is the cycle, not the partner. This is incorporated into schema therapy for couples (Simeone-DiFrancesco et al., 2015), where attention is given to the identification and naming of modes that contribute to the cycle.
(5) **Drama Triangle:** In what Karpman (1968) called the ***Drama Triangle*** (see also Liotti, 2004), dysregulated individuals with disturbed early attachment, become caught up in mode cycles in which they flip between three coping modes: Victim, Rescuer and Perpetrator. These correspond to ***Self-Pity/Victim***, ***Self-Sacrificer/Rescuer***, and ***Bully and Attack/Scolding Overcontroller*** modes. This results in a relationship in which the two parties A and B flip roles chaotically between complementary roles, for example from (1) A: Persecutor with B: Victim, to (2) A: Rescuer with B: Victim, to (3) B: Persecutor with A: Victim, to (4) B: Rescuer with A: Victim. In order to extract themselves from these cycles, individuals need to find a **Healthy Adult** that can step out of them.
(6) **Sources of the Inner Critic:** It is important to recognize that self-critical or demanding voices can have different sources. Initially it is pragmatic to use the term ***Inner Critic*** for them. However, in due course, it can be important to identify the source because this has implications for treatment. When the source is an introjected ***Parent*** voice, we work to identify the source in one or more parent or authority figures and help the child separate from the toxic parent, often by sending the parent away, and initiating a reparenting relationship with the therapist. However, self-critical messages may have their source in the ***Angry Child*** which is directed back at the self, resulting in a ***Flagellating Overcontroller*** as discussed above. In this case we need to validate the experience of the ***Angry Child*** and work with self-compassion (Neff, 2013). A third process involves self-critical messages being recruited by an ***Overcontroller*** mode, particularly a ***Perfectionist*** or ***Eating Disordered Overcontroller***, with the function of motivating individuals to meet the rules or standards built into that mode. In this case the therapeutic task is to change the decision to cope in that way (Edwards, 2017, 2019). This can complement working to send away an introjected parent, or support the ***Angry Child*** in redirecting his/her anger toward its proper target.
(7) **Same behavior, different mode:** A particular behavior may have its source in different modes depending on the underlying motivation. Consider, for example, self-harming (Abbas et al., 2018). Consider how a suicide attempt might arise from several modes, including (1) a ***Detached Self-Soother*** with a desire to end overwhelming distress and find peace; (2) a ***Care-seeking Overcompensator***, a “cry for help,” a way to show people how desperate one feels; (3) an ***Enraged Child*** channeled through ***Bully and Attack*** intending to punish someone, to make them sorry for their behavior, or (4) ***Flagellating Overcontroller*** as a way to punish oneself.

## Conclusion

As indicated at the outset, the aim of this article has been to present a list of schema modes that draws on the existing literature within and beyond schema therapy and is phenomenologically grounded and clinically useful. Experienced schema therapists may have further modes to add, or be able to elaborate on some of the modes listed here that are only briefly described. As it stands, however, the present list provides a comprehensive resource that can alert clinicians to the differentiation of processes that are important for case conceptualization, especially for difficult cases where some of the less familiar modes described here play a role. An additional aim has been to present a number of concepts and accounts of processes that are relevant to differentiating and working with modes in clinical practice. Grounded in the literature but focused on clinical practice and, in particular, the challenges posed by case conceptualization, this article is intended to be a practical resource for clinicians working within the schema therapy model. As a comprehensive review, it is also intended to catalyze further debate and promote research that will contribute to the further differentiation and understanding of schema modes and the dynamic psychological processes that give rise to them.

## Author Contributions

The author confirms being the sole contributor of this work and has approved it for publication.

## Conflict of Interest

The author declares that the research was conducted in the absence of any commercial or financial relationships that could be construed as a potential conflict of interest.

## Publisher’s Note

All claims expressed in this article are solely those of the authors and do not necessarily represent those of their affiliated organizations, or those of the publisher, the editors and the reviewers. Any product that may be evaluated in this article, or claim that may be made by its manufacturer, is not guaranteed or endorsed by the publisher.

## Footnotes

1 The author expresses gratitude to Poul Perris, Vartouhi Ohanian, and Jeff Conway for contributing to discussions which led to this approach.
